# Undertriage in Older Emergency Department Patients – Tilting against Windmills?

**DOI:** 10.1371/journal.pone.0106203

**Published:** 2014-08-25

**Authors:** Florian F. Grossmann, Thomas Zumbrunn, Sandro Ciprian, Frank-Peter Stephan, Natascha Woy, Roland Bingisser, Christian H. Nickel

**Affiliations:** 1 Emergency Department, University Hospital Basel, Basel, Switzerland; 2 Clinical Trial Unit, University Hospital Basel, Basel, Switzerland; University of Granada, Spain

## Abstract

**Objectives:**

The aim of this study was to investigate the long-term effect of a teaching intervention designed to reduce undertriage rates in older ED patients. Further, to test the hypothesis that non-adherence to the Emergency Severity Index (ESI) triage algorithm is associated with undertriage. Additionally, to detect patient related risk factors for undertriage.

**Methods:**

Pre-post-test design. The study sample consisted of all patients aged 65 years or older presenting to the ED of an urban tertiary and primary care center in the study periods. A teaching intervention designed to increase adherence to the triage algorithm. To assess, if the intervention resulted in an increase of factual knowledge, nurses took a test before and immediately after the teaching intervention. Undertriage rates were assessed one year after the intervention and compared to the pre-test period.

**Results:**

In the pre-test group 519 patients were included, and 394 in the post-test-group. Factual knowledge among triage nurses was high already before the teaching intervention. Prevalence of undertriaged patients before (22.5%) and one year after the intervention (24.2%) was not significantly different (χ2 = 0.248, df = 1, p = 0.619). Sex, age, mode of arrival, and type of complaint were not identified as independent risk factors for undertriage. However, undertriage rates increased with advancing age. Adherence to the ESI algorithm is associated with correct triage decisions.

**Conclusions:**

Undertriage of older ED patients remained unchanged over time. Reasons for undertriage seem to be more complex than anticipated. Therefore, additional contributing factors should be addressed.

## Introduction

Undertriage, an assignment of an inadequately low triage level, increases the patients' risk for health status deterioration while waiting. To date, a threshold for undertriage rates is not generally defined in the literature, but to achieve an undertriage rate of less than 10% is recommended [1]. Older ED patients are a vulnerable patient group and are at risk of undertriage [2], [3]. Reasons for this are poorly understood, but likely are multifactorial. The interpretation of vital signs, for instance, is more difficult in older patients, as they may be normal even in serious disease [4]–[6]. Further, older ED patients often present with non-specific complaints such as weakness [7], which may lead to undertriage [8].

However, when correctly applied, the Emergency Severity Index (ESI) proved to be valid and reliable for all ED patients, also in the German version [1], [3], [9], [10]. When using the ESI as a triage instrument, the two main pitfalls of triage in older ED patients appear to be non-adherence to the algorithm and neglect of high risk situations [3]. Presence of a high risk situation or changes in vital signs determines whether a patient can be left waiting or cannot wait (ESI 3 versus 2). This is of importance as delayed care and a prolonged stay in the ED can lead to adverse events e.g. by delaying transfer of critically ill patients to the intensive care unit [11], [12].

Tackling the phenomenon of undertriage therefore might help to detect older ED patients at risk early in the evaluation process. The objective of this study was to test if a teaching intervention had a sustainable long-term effect on reducing undertriage rates in older patients. Further, we aimed to test the hypothesis that non-adherence to the triage algorithm is associated with undertriage. Additionally, we sought to detect patient related risk factors and reasons for undertriage in older ED patients.

## Methods

### Study design

We chose a pre-post-test design. Data of a previous evaluation were used for the pre-test analysis [3]. To investigate if our teaching intervention had a sustainable long-term effect, post-test data were collected one year after the teaching intervention. We deliberately chose a one year interval in order to exclude an intervention effect leading to a mere short term improvement immediately after training.

### Sample and Setting

The samples consisted of consecutive ED patients aged 65 years or older. For the pre-test, we used data from a previous evaluation, which was conducted between April 6^th^ and 27^th^ in 2009. During that period, 519 patients were included [3]. The teaching intervention occurred in March 2010. Sampling for the post-test period was conducted between May 1^st^ and May 17^th^ in 2011. In this period, 505 patients were seen in the ED. Assuming a reduction of the proportion of undertriaged patients from an estimated 22.5% in the pre-test data by half in the post-test data, ca. 230 patients per group (pre- and post-test) would be needed to show a statistically significant difference in proportions with a power of 90% at a significance level of 0.05.

The study was conducted at the ED of the University Hospital Basel, Switzerland, an urban tertiary care center, with an annual census of 42′000 ED visits. Adult patients of all specialties except pediatrics, gynecology and obstetrics, ophthalmology, and odontology are treated in our ED. All patients were triaged using the German translation of the ESI [9]. All triage nurses were either certified in emergency nursing or had longstanding experience in emergency nursing. They trained according to the recommendations of the ESI implementation handbook [1], which is a basic, formal four hour training. In our institution, the triage decisions of newly trained nurses are supervised during the first day of triage. Four education sessions per year including discussion of case scenarios are mandatory for triage nurses.

### Selection of Patients

The study sample consisted of all patients aged 65 years or older presenting to the ED in the study periods. Enrolment was done consecutively. Patients where no triage level was documented were excluded from the analysis.

### Intervention

To increase adherence to the triage algorithm, we designed a teaching intervention to facilitate a correct triage level assignment in older ED patients. The intervention consisted of a 1 hour lecture mandatory for all triage nurses. The content of the training session was based on the results of our former study, specifically addressing the pitfalls [3] (neglect of high risk situations, inadequate interpretation of vital signs). Real, exemplary cases of undertriage were presented and extensively discussed with 2 triage experts (FFG and CHN).

### Methods of Measurement

#### Effect of the teaching intervention on triage nurses' factual knowledge

To test if the teaching session resulted in an increased knowledge on the pitfalls of triage of older patients, participants took a test before and immediately after the teaching session. The test consisted of 6 case scenarios on which the participants had to assign a triage level as well as a multiple choice question on the definition of live saving interventions. The case scenarios were identical before and after the teaching sessions. The participants were blinded to the test results until the second test was completed.

#### Determination of a triage level

The ESI level was determined by triage nurses following the four decision points A to D of the ESI triage algorithm [1]. ESI level 1 is assigned to patients who need an immediate life-saving intervention (decision point A). ESI level 2 represents patients who should not wait because of a high-risk situation, a new onset of confusion, lethargy, disorientation, or severe pain or distress (decision point B). ESI levels 3, 4, and 5 are assigned to patients who need more than one, one, or no resources, respectively (decision point C). Before assigning ESI level 3 to a patient, vital signs must be assessed (decision point D). If they are outside defined limits (heart rate >100/min, respiratory rate >20/min, or oxygen saturation <92 percent), the triage nurse must consider assigning ESI level 2 [1].

#### Inadequate triage

Inadequate triage was defined as incongruence between the triage nurse's ESI assignment and the consensual ESI level retrospectively assigned by two triage experts. Undertriage was defined as cases where the triage nurse's ESI level indicated lower acuity than the experts' ESI level. Overtriage was defined accordingly.

#### Reasons for inadequate triage

Reasons for under- and overtriage were recorded and classified according to the decision points of the ESI algorithm A–D. We coded the reason for undertriage as unknown in cases where the triage decision could not be reconstructed based on the triage nurses' notes. In these four cases the experts' triage designation was based on the available data.

#### Adherence to the triage algorithm

The triage nurse must consider assigning an ESI level 2 if vital signs are in the danger zone (decision point D). Thus, one possibility to measure adherence to the triage algorithm retrospectively is to test whether vital signs are correctly assessed at decision point D. In cases where the algorithm required no vital sign assessment (decision points A, B, and C), adherence could not be measured. Consequently, we defined the triage nurse as being non-adherent if no vital signs were documented at decision point D, as adherent if relevant vital signs were assessed and as fully adherent if a full set of vital signs was documented including heart rate, blood pressure, temperature, oxygen saturation, and respiratory rate.

#### Covariates

Age, gender, and time of arrival were retrieved from the hospital patient database; types of complaints (defined as non-specific, specific, or trauma following a previously published framework [13]) and mode of arrival (walk in or direct ED boarding) were abstracted from patient charts.

### Data collection and processing

Pre-test data were collected in the period from April 6^th^ to April 27^th^ 2009 [3]. The intervention took place in March 2010. For the post test period (from May 1^st^ until May 17^th^ 2011), data were retrieved from patient records. A chart abstractor (SC) presented the triage nurses' notes to two experts (CHN, FFG), who reviewed the triage nurses' notes or handover protocols of direct boarders and independently assigned an ESI level according to the decision points of the ESI algorithm. Assessors were blinded to the ESI level assigned by the triage nurse and to all patient outcomes. This approximates decision-making in a real triage environment, where no additional information beside the patient's history is available. If experts disagreed on an ESI assignment, a consensus was reached by discussing the case. In a second step, charts were analyzed by both triage experts in order to determine reasons for inadequate triage, using a standardized abstraction form.

For data management and analyses, R 3.0.2 [14] and SPSS 21.0 [15] were used. The study was approved by the local ethics committee “Ethikkommission Nordwest- und Zentralschweiz” (EKNZ, identifier No 109/11). Written informed consent was not obtained, as the study was classified as a quality control measure. All data were anonymized and de-identified before analysis.

### Primary data analysis

To evaluate if the teaching intervention had an immediate effect on factual knowledge, a paired Wilcoxon signed ranks test was used comparing the results of the test before and immediately after the teaching session. To test the long-term effect of our teaching intervention on undertriage, we compared prevalence rates for undertriage in the pre- with the post-test period. We used the Pearson's χ^2^ test with Yate's continuity correction. The same test was used to compare undertriage rate per ESI level between pre- and post-test. Risk factor analysis was performed by fitting simple logistic regression models for the response “undertriage” and a series of candidate risk factors in the pre- and post-test groups as well as in the combined data set. To illustrate age dependency of undertriage, probability density estimates of the age of undertriaged patients relative to the probability density estimates of age of all patients were calculated. To test whether adherence has an effect on correct triage, logistic regression models for the response “correct triage” were fit for the case that some vital signs were assessed at triage (adherence) and for the case that vital signs including respiratory rate were assessed (full adherence).

## Results

### Sample characteristics

In the pre-test group, 519 patients were included [3]. In the post-test period, 511 patients aged 65 years or older were treated in the ED. Of these, 117 patients had to be excluded. Of these 3 patients were referred from other hospitals, 6 records were not available, and 108 patients had no ESI level assigned (of which 65 were direct-to-bed patients, and 43 for unknown reason),; In the final analysis, 394 patients were included. The time period needed to include approximately 500 consecutive older ED patients was shorter in the post-test-period, reflecting an increase in ED census.

**Figure 1:**
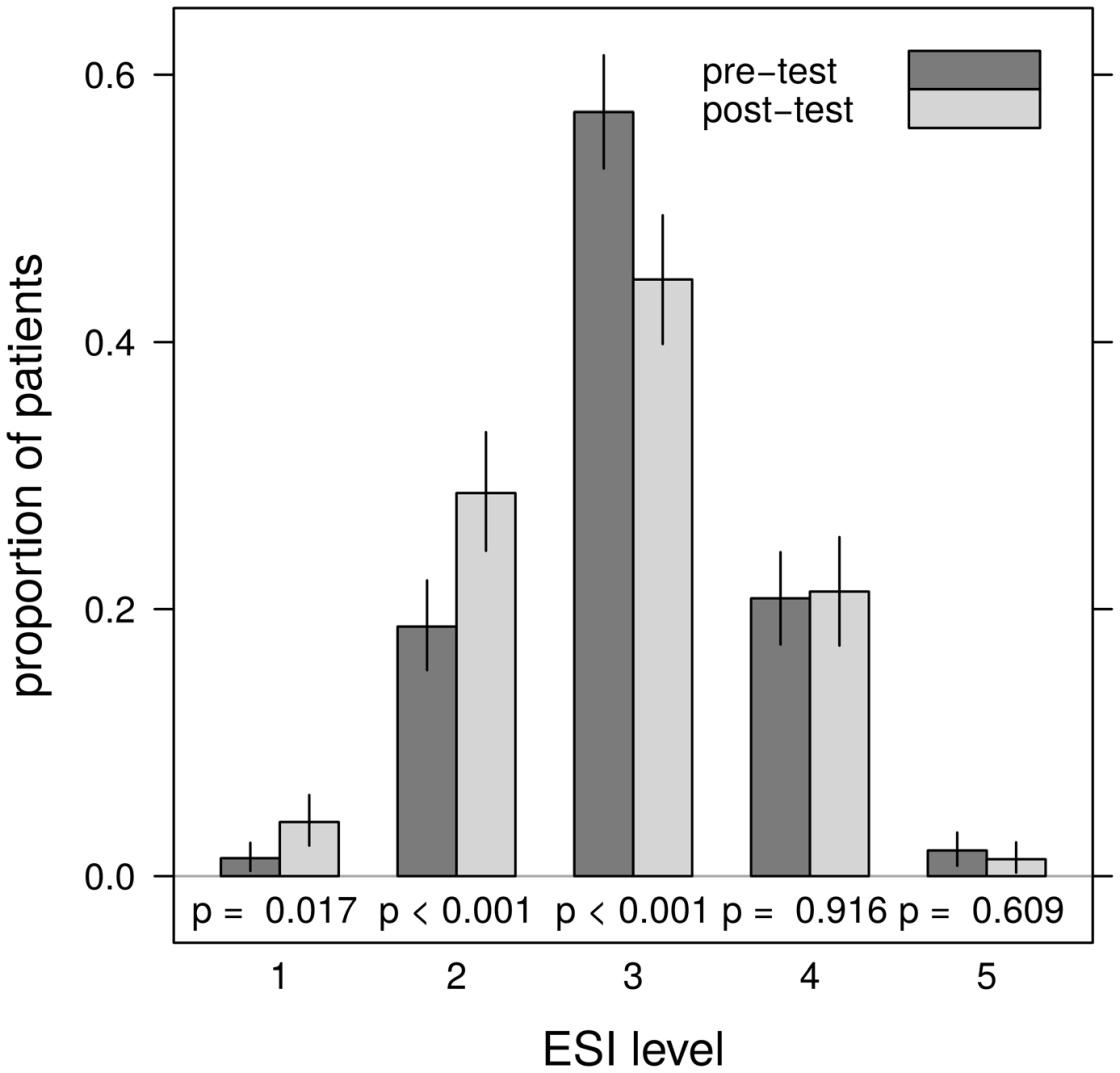
Distribution of ESI triage level proportions with estimated 95% confidence interval. The p values are from the corresponding Pearson's χ2 test with Yate's continuity correction.

Characteristics of both groups are shown in table 1: The proportion of ESI levels differs significantly between the pre- and the post-test period for ESI level 1, 2, and 3 (Pearson's χ^2^ test with Yate's continuity correction) with ESI 1 and 2 being more and ESI 3 being less prevalent in the post-test period (figure 1).

**Table 1:** Sample characteristics.

	Pre-test(N = 519)	Post-test(N = 394)	p-value
Age (years)	72/79/84	71/78/84	
Sex, male	45.9% (237)	46.2% (182)	
ESI-level by triage nurse			
1	1.3% (7)	4.1% (16)	0.017
2	18.7% (97)	28.7% (113)	<0.001
3	57.2% (297)	44.7% (176)	<0.001
4	20.8% (108)	21.3% (84)	0.916
5	1.9% (10)	1.3% (5)	0.609
Inadequate triage			0.619
Undertriage	22.5% (117)	24.1% (95)	
Overtriage	2.9% (15)	3.6% (14)	
Type of complaint (ESI 2 and 3 only)	(N = 394)	(N = 289)	0.057
Non-specific	10.2% (40)	15.6% (45)	
Specific	71.1% (280)	69.9% (202)	
Trauma	18.8% (74)	14.5% (42)	

a/b/c represent the lower quartile a, the median b, and the upper quartile c for continuous variables.

n is the number of non-missing values.

Numbers after percentages are frequencies.

Patients were triaged by 16 different nurses in the pre-test period, and 17 nurses in the post-test period respectively. Due to shift planning, 3 nurses, who triaged in the pre-test period were not on duty in the post-test period, and four nurses were new triage team members at the time the intervention took place. All triage nurses who participated in the post-test period had received a teaching intervention. Of these 17 nurses, 11 nurses participated in the teaching session as described, and 6 were trained individually.

### Prevalence of and reasons for undertriage

The difference between the prevalence of undertriaged patients before (22.5%) and after the intervention (24.2%) was not significant (χ^2^ = 0.227, df = 1, p = 0.634. The proportions of undertriage were compared per ESI level. None of the five comparisons resulted in a significant difference (ESI 1: no comparison possible because there were no undertriaged patients, ESI 2: p = 1.000, ESI 3: p = 0.198, ESI 4: p = 0.281, ESI 5: p = 0.333). Reasons for undertriage are shown in table 2.

**Table 2:** Reasons for undertriage (according to the decision points A-D of the ESI triage algorithm).

Reason	Pre-test	Post-test
	Frequency (%)	Frequency (%)
Life-saving intervention required (A)	4 (3.4)	2 (2.1)
High risk situation (B)	29 (24.8)	22 (24.2)
Confused/lethargic/disoriented (B)	3 (2.6)	3 (3.2)
Severe pain/distress (B)	17 (14.5)	10 (10.5)
Resources (C)	25 (21.4)	24 (25.3)
Vital signs in danger zone (D)	20 (17.1)	8 (8.4)
Severe pain/distress + vital signs in danger zone (B+D)	3 (2.6)	3 (3.2)
High risk situation + vital signs in danger zone (B+D)	4 (3.4)	17 (17.9)
Severe pain/distress + high risk situation (B+D)	3 (2.6)	2 (2.1)
Confused/lethargic/disoriented + vital signs in danger zone (B+D)	1 (0.9)	0 (0.0)
Unknown	8 (6.8)	4 (4.2)
Total	117 (100)	95 (100)

### Effect of the teaching intervention on factual knowledge

Of the 11 eleven triage nurses, who participated in the teaching session, ten took the test before and after the lecture. One nurse had to leave the session before the second test. The six nurses who were trained individually did not take the test. An increase of factual knowledge could not be detected; the mean results were 4.9 points before and 5.3 points (compared to a maximum of 6) after the lecture. The difference for the 10 available pairs was not significant (Wilcoxon signed ranks test, with continuity correction, V = 16.5, p = 0.492).

### Interrater reliability of expert ratings

Interrater reliability testing resulted in high agreement between the two experts: Raw agreement was 0.893 (95% CI 0.863–0.924), Cohen's weighted kappa was 0.908 (95% CI 0.877–0.941), and Spearman's rho was 0.908 (0.875–0.942).

### Risk factors for undertriage

**Figure 2:**
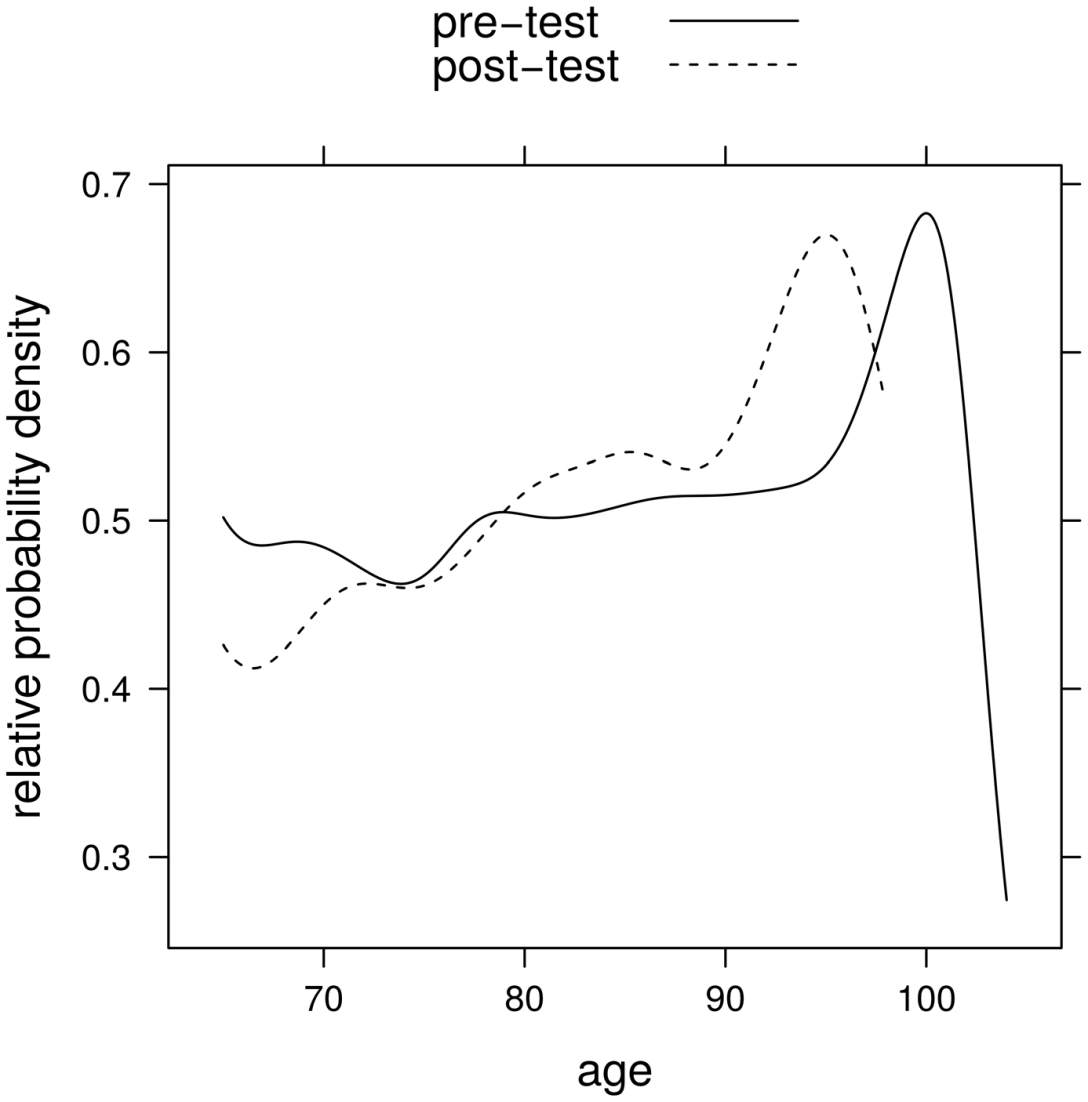
Estimated proportions of patients with undertriage in the pre- and post- treatment groups. The proportions are given as ratios of the kernel probability density estimate of age of patients with undertriage and the kernel probability density estimate of age of all patients.

Sex, age, mode of arrival, and type of complaint considered individually had no significant effect on undertriage (logistic regression models, Table 3). However, when looking at the relative probability density estimates (probability density estimates of age of undertriaged patients relative to probability density estimates of age of all patients) undertriage seems to increase with age in the post treatment group (Figure 2).

**Table 3:** Results of simple logistic regression models for undertriage on risk factors. Intercepts (odds) are not shown.

Model term	Estimate (95% CI)	p value
Sex: male – female (OR), pre-test group	1.03 (0.68–1.56)	0.879
Sex: male – female (OR), post-test group	0372 (0.45–1.15)	0.165
Sex: male – female (OR), combined data set	0.88 (0.65–1.20)	0.428
Age: per year (OR), pre-test group	1.01 (0.98–1.03)	0.702
Age: per year (OR), post-test group	1.03 (1.00–1.06)	0.095
Age: per year (OR) combined data set	1.01 (0.99–1.03)	0.170
Admission mode*: direct boarder – walk-in (OR)	0.92 (0.65–1.52)	0.758
Presenting complaint*: nsc – specific (OR)	1.66 (0.77–3.60	0.197
Presenting complaint*: nsc – trauma (OR)	1.18 (0.47–2.91)	0.727
Presenting complaint*: nsc – specific/trauma/other(OR)	0.76 (0.07–7.11)	0.809

^*^ The variables “admission mode” and “presenting complaint” were collected in the post test group only.

nsc, non-specific complaint.

### Adherence

Of the 176 patients with ESI level 3 assigned by the triage nurses, 156 patients (88.6%) had relevant vital signs assessed at triage, reflecting adherence according to our definition. Full adherence is given when a full set of vitals was assessed, which was only the case in 28 patients (15.9%). Assessment of respiratory rate was significantly associated with correct triage decisions (Table 4).

**Table 4:** Logistic regression for correct triage on the predictors “vital signs” and “respiratory rate” which serve as surrogate for adherence, and full adherence, respectively.

Model term	Estimate (95% CI)	p-value
Vital signs at triage – later (OR)	0.93 (0.32–2.74)	0.898
Respiratory rate not assessed – assessed (OR)	4.52 (1.99–10.27)	<0.001

(post treatment group, N = 174).

## Discussion

The rates of undertriage were stable over time in our study. The teaching intervention geared towards adherence to the ESI algorithm did not result in a sustainable decrease of undertriage rates one year after a teaching intervention. Factual knowledge was consistently high before and immediately after the intervention suggesting that undertriage is not merely a matter of factual knowledge. Independent risk factors for undertriage could not be detected, but a trend towards an increase of undertriage rates with advancing age was observed. Full adherence to the triage algorithm was rare, but if respiratory rate was assessed, this was associated with correct triage decisions.

Undertriage affects patient safety, but it is poorly understood and rarely studied in the clinical ED setting [2], [3], [16]. Despite the paucity of research in this field, geriatric-specific enhancements to the ESI triage algorithm have been suggested [17]. Other studies found that the ESI algorithm itself may be inaccurate in identifying life threatening conditions in older patients [16].

We are not aware of any study that tested interventions dedicated to reduce undertriage (in older ED patients). Our intervention focussed on adherence to the existing ESI triage algorithm. We showed that adherence in terms of measuring respiratory rate is associated with correct triage decisions. However, respiratory rate was only rarely assessed, although its measurement is mandatory in the algorithm at decision point D. Vital sign assessment helps identifying those patients who are in need of more resources and cannot wait. As vital signs are predictive of patient outcomes, complete vital sign assessment at decision point D is of importance [18]. Inconsequent measurement of respiratory rates in our study is a finding that is in line with the literature [19]. Since correct assessment of respiratory rate takes 60 seconds, triage nurses might decide to omit respiratory rate assessment in favour to a more rapid workflow. In addition, the respiratory rate is the only vital sign that cannot be measured with devices and might therefore be not performed. This is despite the fact that respiratory rate has additional diagnostic value in a variety of clinical situations and is incorporated in clinical risk scores and guidelines [20]–[22].

Another issue determining whether a patient can wait or cannot wait is the presence of a high risk situation. High risk situations can be identified by certain complaints, red flag signs, or the medical history. Given the high level of factual knowledge as tested with the present study, one might speculate that triage nurses consciously, against their better knowledge, chose to deliberately deviate from the triage protocol. Especially when triage nurses feel as gate-keeper of a crowded ED, they are reluctant to assign ESI level 2 to patients because immediate placement in the ED treatment area is required [1]. Therefore, environmental factors like ED crowding may contribute to higher rates of undertriage which is supported by a qualitative study on triage decision making [23].

Further, triage decision making in our study might have been biased. Although age did not reach statistical significance in the risk factor analysis – possibly due to small sample size in the very old age groups – there seems to be a trend towards undertriage with increasing age.

We learned from our study that more efforts than a single teaching intervention are needed to overcome undertriage in older ED patients. As triage decisions are made by a heterogeneous collective of triage nurses, individual triage performance should be monitored. Low performers may profit from interventions tailored towards their needs.

To explain the stability of undertriage pattern over time, several contributing factors can be considered. Obviously, factual knowledge about the ESI triage algorithm does not necessarily translate into triage nurses' adherence in a real triage environment. We hypothesize that awareness of the special care needs of older ED patients, of potential age-bias, and of environmental factors might have a positive impact on adherence to the triage algorithm. Further research, such as qualitative studies might help to understand triage decision making in this vulnerable patient group. To confirm that undertriage rates increase with advancing age, quantitative studies with larger sample sizes in the oldest age groups should be performed.

### Limitations

The quasi experimental pre-post-test design of this single center study is subject to unmeasured differences between the two periods. A further limitation is the retrospective triage level assignment by the experts. However, retrospective chart review is an accepted method to determine triage quality [1].

In a group of patients, no triage level was documented leading to possible selection bias, which is reduced by consecutive sampling. Additionally, changes in the ED patient population with higher acuity patients and higher overall ED census in the post-test period might have influenced undertriage rates.

Lastly, the list of potential risk factors for undertriage may have been incomplete. We did not assess for association of undertriage with other potential risk factors such as dementia, residence in a nursing home, or do not resuscitate (DNR) orders leading to the possibility of confounding and bias.

### Conclusion

Misapplication of existing triage criteria is an important reason for undertriage in older patients when applying the ESI. Apart from non-adherence, reasons for undertriage in older ED patients appear to be more complex than anticipated. Improving awareness of additional contributing factors such as age bias might have a positive impact on triage decision-making.
